# Multifunctionality in Lead-Free Rb_2_XTaBr_6_ (X = Na, Li) Double Perovskites: A First-Principles Study of Structural, Elastic, Optoelectronic, and Thermoelectric Properties

**DOI:** 10.3390/nano16181153

**Published:** 2026-09-14

**Authors:** Fareesa Tasneem Tahir, Arzoo Hassan, Xiao-Qing Tian, Qing-Feng Sun, Zhi-Rui Gong, Ya-Dong Wei

**Affiliations:** 1 College of Physics and Optical Engineering, State Key Laboratory of Radio Frequency Heterogeneous Integration, Shenzhen University, Shenzhen 518060, Chinagongzr@szu.edu.cn (Z.-R.G.); ywei@szu.edu.cn (Y.-D.W.); 2 National Laboratory of Solid State Microstructures, Nanjing University, Nanjing 210093, China; 3 International Center for Quantum Materials, School of Physics, Peking University, Beijing 100871, China; 4 Hefei National Laboratory, Hefei 230088, China

**Keywords:** magnetic materials, optoelectronics, first principles, double halide perovskites

## Abstract

First-principles FP-LAPW calculations were used to investigate the structural, mechanical, electronic, optical, and thermoelectric properties of the double halide perovskites Rb_2_NaTaBr_6_ and Rb_2_LiTaBr_6_. Structural optimization confirmed their stable cubic phase. Elastic analysis shows they are mechanically stable, ductile, and anisotropic. Spin-polarized calculations reveal that both compounds are half-metallic ferromagnets with an integer total magnetic moment of 2 μB per formula unit, dominated by the Ta-d states. They exhibit strong dielectric response and strong UV light absorption originating from interband electronic transitions, despite their intrinsic metallic ground state. Boltzmann transport theory revealed a temperature-dependent enhancement of electrical conductivity and power factor. Both materials, especially Rb_2_LiTaBr_6_, show promise for high-temperature thermoelectric and UV optoelectronic applications.

## 1. Introduction

This tolerance underlies real applications in optoelectronics, energy conversion, thermoelectrics, and neuromorphic electronics, driven by strong light absorption, tunable band gaps, and processing routes that are often cheaper than those required by conventional semiconductors [1,2]. Over the past decade, this combination of tunability and low cost has made perovskites among the most heavily studied classes of functional materials. Not every chemically interesting cation is compatible with the single-perovskite B-site: an unsuitable ionic radius, a coordination geometry incompatible with the octahedral environment, or an oxidation state that is unstable under operating conditions can each preclude incorporation. Mixed or fluctuating oxidation states typically compromise long-term stability, accelerating degradation under heat, humidity, or light exposure [3,4]. A material with strong initial performance but rapid failure under ambient conditions offers little practical value, and much of the perovskite literature addresses precisely this trade-off between performance and durability. Double perovskites, A_2_BB′X_6_, relax this constraint by distributing the B-site charge between two ordered cations. This doubles the unit cell and substantially expands the accessible compositional space relative to the single-perovskite framework because structural and electronic demands are shared between two cations, chemistries that are unstable as single-cation B-sites can often be stabilized in combination, multiplying the number of viable element pairings [5,6]. Double perovskites are consequently more stable on average, while offering additional degrees of freedom for engineering electronic, optical, and magnetic properties through independent choice of the B and B′ cations [7]. Cs_2_AgBiBr_6_ remains the most extensively studied double-perovskite semiconductor [8]. Single crystals show a room-temperature indirect band gap of 1.95 eV and a photoluminescence lifetime of up to 660 ns, and the compound can be solution-processed, keeping fabrication costs low [9]. These properties made it a benchmark compound for the field. However, its indirect gap requires phonon-assisted optical transitions, weakening absorption near the band edge; reported solar-cell efficiencies for Cs_2_AgBiBr_6_ devices remain in the low single digits regardless of device optimization [10,11,12]. Systematic studies of Cs_2_AgBiBr_6_- and Cs_2_NaGaX_6_ (X = Cl, Br)-related A_2_BB′X_6_ compounds have established consistent structure–property relationships: substituting the B or B′ cation shifts the band character in predictable ways, octahedral tilting and distortion correlate with measurable changes in band gap and carrier mobility, and these same structural parameters feed directly into the Seebeck coefficient and electrical conductivity that determine thermoelectric performance [13,14,15,16]. Composition, more than processing conditions or defect engineering, therefore emerges as the principal lever for tuning performance in this materials class.

Despite this activity, coverage of the double-perovskite compositional space remains incomplete. Many promising B′ chemistries, particularly transition metals and heavy elements with strong spin–orbit coupling or partially filled d-shells, remain untested or have only been examined superficially [17]. Modeling transition-metal double perovskites accurately is nontrivial: the localized d-electron states involved behave very differently from the delocalized s and p states that dominate simpler perovskite compositions, and the plain generalized gradient approximation (GGA) used in most density-functional-theory (DFT) calculations systematically underestimates electron correlation in these localized orbitals [18,19]. The resulting error understates band gaps, sometimes to the point of predicting an artificial metal and overstates electron delocalization, a well-documented failure mode that propagates into every downstream property, from optical absorption to thermoelectric transport [20,21]. The Dudarev implementation of the on-site Hubbard correction (GGA + U) addresses this by penalizing unphysical delocalization of the correlated d-orbitals and has a long track record of improving predicted band structures, densities of states, and optical/transport properties across the halide-perovskite and transition-metal oxide/halide literature. For a system built around a transition metal at the B′ site, using GGA + U rather than plain GGA is a methodological requirement rather than a technical choice [22,23].

This kind of transition-metal d-orbital-driven band reorganization is well documented in other host lattices. In Ni-doped CeO_2_, for example, DFT calculations show that Ni-3d states introduced into the CeO_2_ band structure narrow its gap and lower the Fermi level because Ni enters only as a dilute dopant; however, these new states remain localized, defect-like levels within an otherwise intact 3 eV gap, narrowing but not closing it. As detailed in Section 3.3, a fully ordered, stoichiometric d-electron cation produces a qualitatively stronger effect: Ta-5d states in Rb_2_NaTaBr_6_ and Rb_2_LiTaBr_6_ form a dispersive, itinerant band spanning the entire Brillouin zone, driving complete closure of the gap (Eg = 0) rather than a merely reduced one [24].

Tantalum-based double perovskites, in which Ta occupies the B′ site, have received almost no attention in the literature to date, despite tantalum’s favorable combination of high oxidation state, strong spin–orbit coupling, and chemical robustness. We address this gap with a first-principles study of two previously unreported compositions, Rb_2_NaTaBr_6_ and Rb_2_LiTaBr_6_, using density functional theory within the GGA + U framework. The work has four aims. First, to establish the structural stability and elastic properties of both compounds to confirm mechanical feasibility. Second, to determine the spin-polarized electronic band structure and density of states for each compound to establish whether the ground state is metallic or semiconducting, rather than assuming a band gap by analogy with systems such as Cs_2_AgBiBr_6_, and quantify the resulting spin magnetic moments and magnetic ground state. Third, to evaluate key optical properties, including the dielectric function, absorption coefficient, and refractive index, focusing on the ultraviolet interband response relevant to UV optoelectronic applications rather than visible-light photovoltaics. Fourth, to calculate thermoelectric parameters, the Seebeck coefficient, electrical conductivity, and power factor, as a function of temperature at the intrinsic Fermi level.

Several recent first-principles studies provide direct benchmarks for the present work, including halide A_2_BBiCl_6_ (A = Cs, K; B = Ag, Au), X_2_YBiCl_6_ (X = Cs, Na; Y = Ag, Au), cation-tuned Sr_2_MReO_6_ (M = Li, Na, K), cubic X_2_YInO_6_ (X = Ba, Sr; Y = Nb, V), and X_3_NI_3_ (X = Sr, Ba) perovskites, each investigated for structural, electronic, optical, elastic, and thermoelectric behavior [25]. Together, these results indicate whether Rb_2_NaTaBr_6_ and Rb_2_LiTaBr_6_ are worth pursuing as candidate materials for optoelectronic and thermoelectric applications, and more broadly whether tantalum-based double perovskites merit the same attention already given to their bismuth- and antimony-based counterparts.

## 2. Computational Work

Structural, electronic, optical, mechanical, and thermoelectric properties of Rb_2_NaTaBr_6_ and Rb_2_LiTaBr_6_ were calculated with the full-potential linearized augmented plane wave (FP-LAPW) method in WIEN2k, using the PBE-GGA exchange-correlation functional. Structures were fully relaxed (lattice parameters and internal coordinates), SCF converged to 0.001 Ry, with RMT × Kmax = 6.0 and a Γ-centered Monkhorst–Pack mesh of 2000 k-points in the full Brillouin zone [26]. Convergence in RMT × Kmax (4.0–8.0) and k-mesh density (500–2500 points) was verified to within 0.0005 Ry. Muffin-tin radii (Shannon ionic radii, non-overlapping): Rb 2.50, Na 2.00, Li 1.75, Ta 2.20, Br 2.30 a.u. A Hubbard correction (GGA + U, Dudarev) was applied to the Ta-5d states: U = 0.20 Ry (2.7 eV), J = 0, a value lower than typical oxide U (3–6 eV), reflecting weaker halide covalency. Confirmed by a sensitivity sweep (U = 0–0.30 Ry), both compounds stay metallic throughout, but U = 0.20 Ry best balances correcting GGA’s over-delocalization against physically reasonable Ta-d/Br-p covalency (U = 0.30 Ry over-localizes and suppresses it). Elastic constants (C_ij_) were obtained by the finite-strain method via IRelast; directional Young’s modulus, shear modulus, Poisson’s ratio, and anisotropy were then derived from C_ij_ using ELATE. Optical properties used the linear-response formalism in WIEN2k OPTIC module, interband contributions only, no Drude term, so the 3.5–4 eV absorption onset reflects interband transitions, not a semiconducting gap. Thermoelectric coefficients were computed with BoltzTraP (intrinsic Fermi level, rigid-band approximation); cubic symmetry makes the transport tensors isotropic (σ/τ, κ_e_/τ). S is τ-independent; absolute conductivity, power factor, and ZT assume τ = 1 × 10^−14^ s. ZT = S^2^σT/κ uses electronic κ only (no lattice κ computed), an upper-bound estimate, and increasingly overestimated at high T as the constant-τ assumption weakens [27,28].

## 3. Result and Discussion

### 3.1. Structure Properties

**Figure 1:**
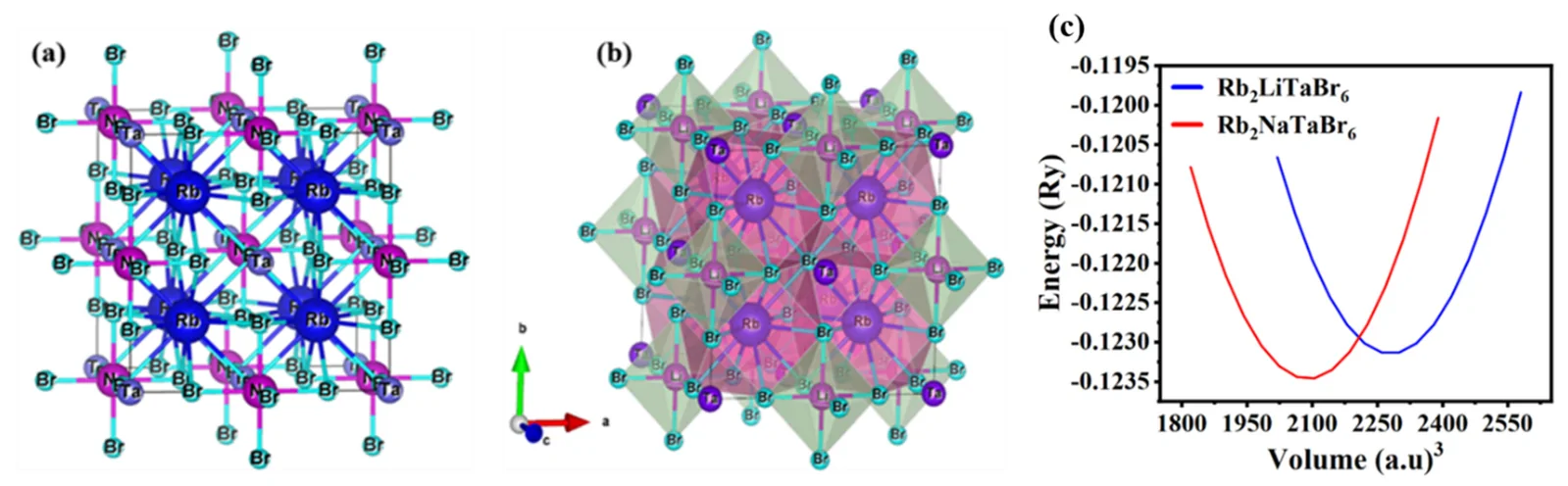
Schematic representation of cubic perovskite structure of (**a**) Rb_2_NaTaBr_6_ and (**b**) Rb_2_LiTaBr_6_. (**c**) Variation in formation energy as a function of unit-cell volume for Rb_2_NaTaBr_6_ and Rb_2_LiTaBr_6._

Rb_2_NaTaBr_6_ and Rb_2_LiTaBr_6_ crystallize in a stable cubic A_2_BB′X_6_ double-perovskite structure, with Rb occupying the A site, Ta occupying the B site and forming corner-sharing TaBr_6_ octahedra, and Na or Li occupying the B′ site, as depicted in Figure 1a,b. This three-dimensional network of corner-sharing TaBr_6_ octahedra provides the structural framework of the lattice. Substituting Na with the smaller Li^+^ ion produces a clear lattice contraction, the optimized lattice constant decreases from 11.08 Å to 10.80 Å, and the equilibrium unit-cell volume decreases correspondingly from 2275.52 a.u.^3^ to 2101.19 a.u.^3^, a = 7.7% volume reduction, consistent with the 2.5% linear contraction expected for a cubic cell (ΔV/V = 3Δa/a). Under the ideal ionic description, charge neutrality requires 2(Rb^+^) + X^+^ + Ta^n+^ + 6(Br^−^) = 0, giving n = +3, i.e., Ta^3+^. The Goldschmidt tolerance factor, t = (rA + rX)/[√2(rB, avg + rX)], and the octahedral factor, μ = rB, avg/rX, were evaluated using Shannon effective ionic radii consistent with this Ta^3+^ assignment: rRb = 1.72 Å (XII-coordinate), rNa = 1.02 Å, rLi = 0.76 Å, rTa^3+^ = 0.64 Å, and rBr = 1.96 Å (VI-coordinate), with rB, avg = (rB′ + rTa)/2. This gives t = 0.919, μ = 0.444 for Rb_2_NaTaBr_6_ and t = 0.964, μ = 0.378 for Rb_2_LiTaBr_6_. Both values fall within the 0.8–1.0 range generally associated with cubic double-perovskite formability, consistent with the cubic phase obtained here by full structural relaxation. This ionic-size-based prediction is independently corroborated by the elastic-constant analysis in Section 3.2, where both compounds satisfy the Born mechanical stability criteria for a cubic lattice. A well-defined minimum in the energy–volume curve further confirms that both compounds are locally stable within the assumed cubic Fm-3m model at their calculated equilibrium volumes [29]. The formation energy was calculated using Equation (1):

(1) $$E_{f}=\;E_{tot}-[2E_{\left(Rb\right)}+E_{\left(Na\right)}+E_{\left(Ta\right)}+6E_{\left(Br\right)}]\;$$

The formation energy (E_f_) was calculated as the difference between the total energy of the compound (E_tot_) and the sum of the total energies of its constituent elements in their respective cubic phases, weighted by their stoichiometric coefficients in the formula unit (Figure 1c). A negative E_f_ indicates that the compound is thermodynamically stable (exothermic formation) relative to its constituent elements, yielding −0.1235 Ry and −0.1234 Ry for Rb_2_LiTaBr_6_ and Rb_2_NaTaBr_6_, respectively, confirming the thermodynamic stability of both compounds. The same Na → Li trend carries over to the mechanical response: Rb_2_LiTaBr_6_ has a higher bulk modulus than Rb_2_NaTaBr_6_, consistent with its more contracted lattice being harder to compress and with stronger interatomic interactions in the Li-based compound. The minimum sits at the equilibrium volume, with curvature on the compression side reflecting repulsive interactions and curvature on the expansion side reflecting the tensile response of the lattice, the steeper curvature for Rb_2_LiTaBr_6_ is consistent with its higher bulk modulus. In general, Na → Li substitution contracts the lattice because of the smaller ionic radius of Li^+^ relative to Na^+^, and this contraction correlates with increased mechanical rigidity without altering the underlying cubic double-perovskite structure. For comparison, structural parameters of the related double perovskite K_2_TlAsX_6_ (X = Cl, Br) are provided in Supplementary Materials.

### 3.2. Elastic and Mechanical Properties

**Figure 2:**
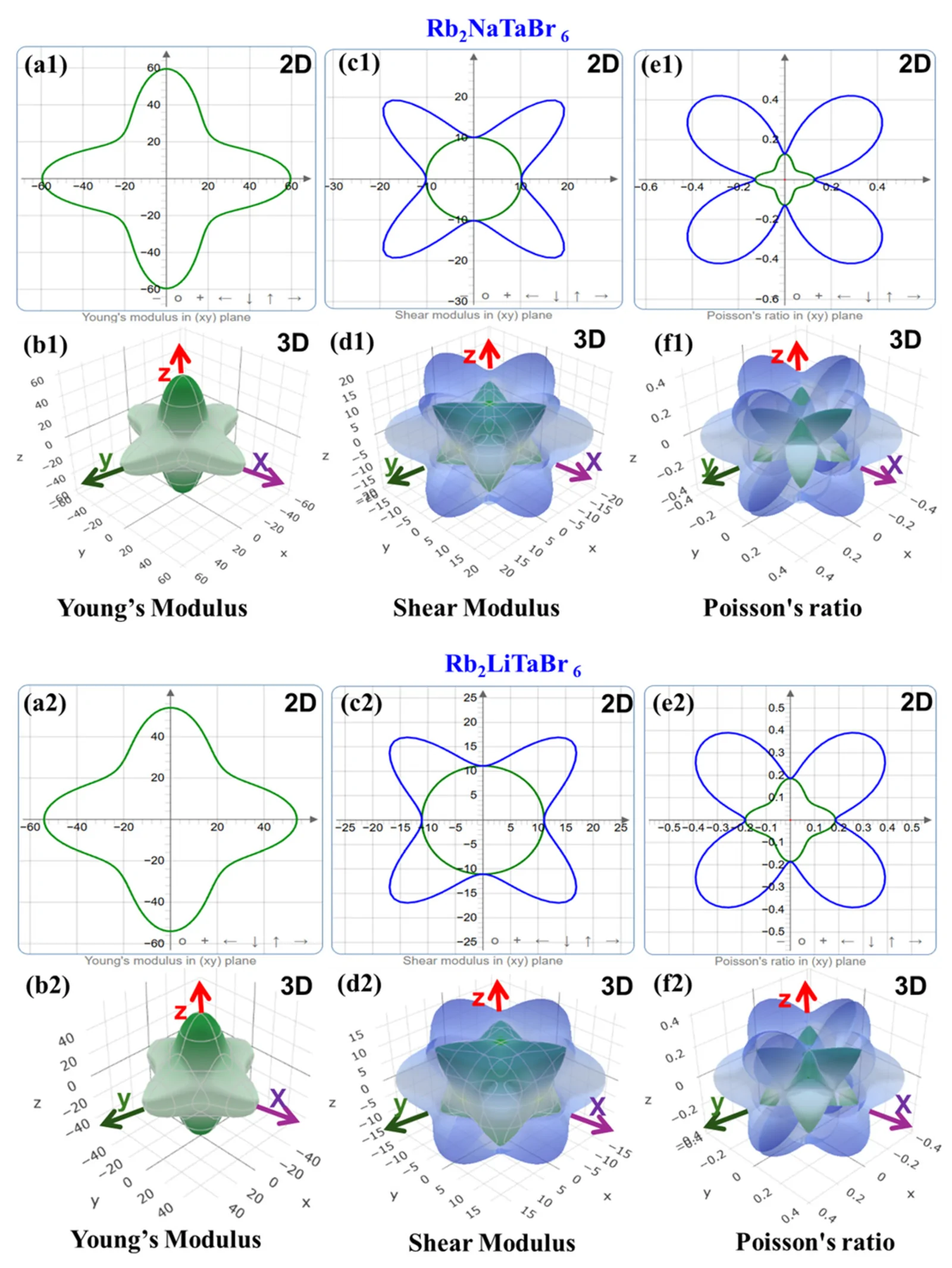
Elastic anisotropy of Rb_2_NaTaBr_6_ (**a1**,**c1**,**e1**) and Rb_2_LiTaBr_6_ (**a2**,**c2**,**e2**) Two-dimensional representations of Young’s modulus, shear modulus and Poisson’s ratio along the x, y and z directions. (**b1**,**d1**,**f1**) and (**b2**,**d2**,**f2**) Corresponding three-dimensional surfaces showing the directional dependence of these elastic properties.

Elastic behavior describes how a solid resists applied stress and recovers its shape once that stress is removed, governed by the elastic constants (C_ij_), which relate stress to strain via Hooke’s law and serve as indicators of strength, hardness, and structural stability. Because Rb_2_NaTaBr_6_ and Rb_2_LiTaBr_6_ crystallize in the cubic phase, only three independent constants, C_11_, C_12_, and C_44_, fully characterize their elastic behavior (Figure 2). Figure 2(a1–f1) describe the elastic behaviors of Rb_2_NaTaBr_6_ and Figure 2(a2–f2) exhibits the same phenomenon for Rb_2_LiTaBr_6_ compounds. These constants, obtained from DFT calculations, are given in Supplementary Materials along with the derived mechanical parameters. Both compounds satisfy the Born mechanical stability criteria for a cubic lattice (C_11_ > 0, C_44_ > 0, C_11_ − C_12_ > 0, C_11_ + 2C_12_ > 0), confirming the stability of their cubic phases. From these constants, the bulk modulus (B), shear modulus (G), and Young’s modulus (E) were calculated using the standard cubic-elasticity relations below.

(2) $$G=\frac{G_{V}+G_{R}}{2},\;B=\frac{C_{11}+2C_{12}}{3},\;E=\frac{9BG}{3B+G},$$

where

(3) $$G_{R}=\frac{5C_{44}\left(C_{11}-C_{12}\right)}{4C_{44}+3\left(C_{11}-C_{12}\right)},\;G_{V}=\frac{C_{11}-C_{12}+3C_{44}}{5},$$

The bulk modulus of Rb_2_LiTaBr_6_ is calculated to be higher than that of Rb_2_NaTaBr_6_ (Table 1), indicating that the Li-based compound is more resistant to volume compression, consistent with its smaller equilibrium volume and stronger interatomic bonding discussed above. The shear modulus values likewise indicate that Rb_2_LiTaBr_6_ is mechanically stiffer than Rb_2_NaTaBr_6_. Based on the following relations, the elastic anisotropy factor and Poisson ratio are then calculated.

(4) $$v=\frac{3B-2G}{2\left(2B+G\right)},\;A=\frac{2C_{44}}{C_{11}-C_{12}}.$$

Both perovskites satisfy the Born mechanical stability criteria for a cubic lattice, confirmed by positive elastic constants (C_11_, C_44_, C_11_ − C_12_, C_11_ + 2C_12_). Poisson’s ratio (ν = 0.354 for Rb_2_NaTaBr_6_, 0.375 for Rb_2_LiTaBr_6_) exceeds the ν = 0.33 brittle/ductile threshold for both, consistent with the Pugh ratio B/G (3.08 and 3.68, both above the B/G = 1.75 threshold), confirming ductile behavior. The Cauchy pressure is positive for Rb_2_LiTaBr_6_ (+2.40 GPa) but slightly negative for Rb_2_NaTaBr_6_ (−1.08 GPa), so Rb_2_NaTaBr_6_ sits near the ductile/brittle boundary while Rb_2_LiTaBr_6_ is ductile by every index. The Zener anisotropy factor (A = 0.386 and 0.486, both well below the isotropic value of 1) confirms both compounds are elastically anisotropic, with Rb_2_NaTaBr_6_ the more anisotropic of the two, as shown in Supplementary Materials. Direction-dependent elastic behavior, mapped via 2D and 3D representations of Young’s modulus, shear modulus, and Poisson’s ratio, confirms this anisotropy structurally: the Young’s modulus is highest along the main crystallographic axes and lowest along the diagonals, consistent with the TaBr_6_ framework bonding more strongly along the principal directions. The shear modulus shows comparable directional spread. Poisson’s ratio is more directionally uniform in Rb_2_NaTaBr_6_, whereas Rb_2_LiTaBr_6_ shows a region of negative Poisson’s ratio, indicating orientation-dependent auxetic behavior under tensile load.

### 3.3. Electrical Properties

**Figure 3:**
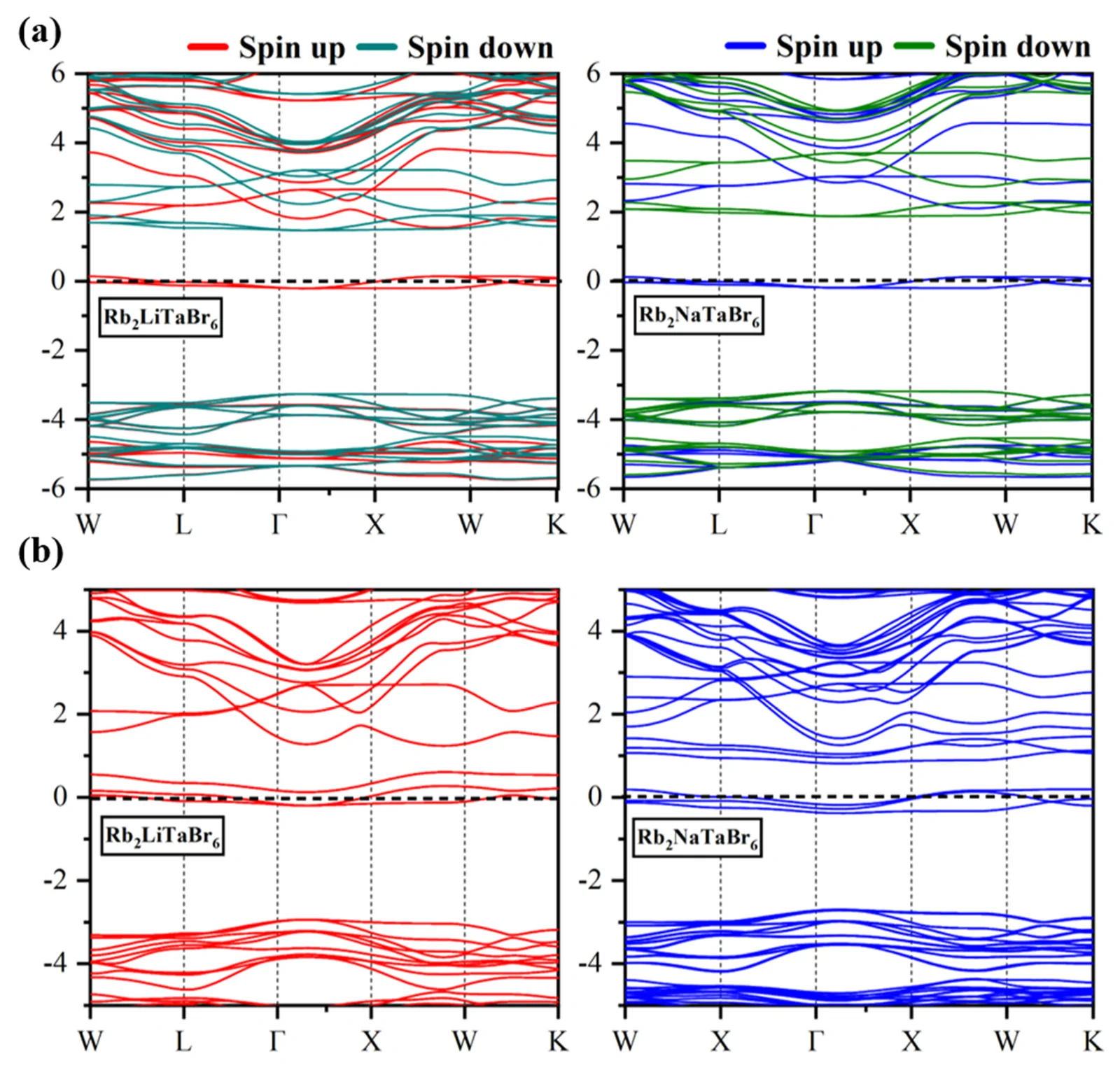
(**a**) Spin-polarized band structures and (**b**) Spin–orbit coupling (SOC) of Rb_2_NaTaBr_6_ and Rb_2_LiTaBr_6_ calculated using the GGA + U method along high-symmetry directions.

**Figure 4:**
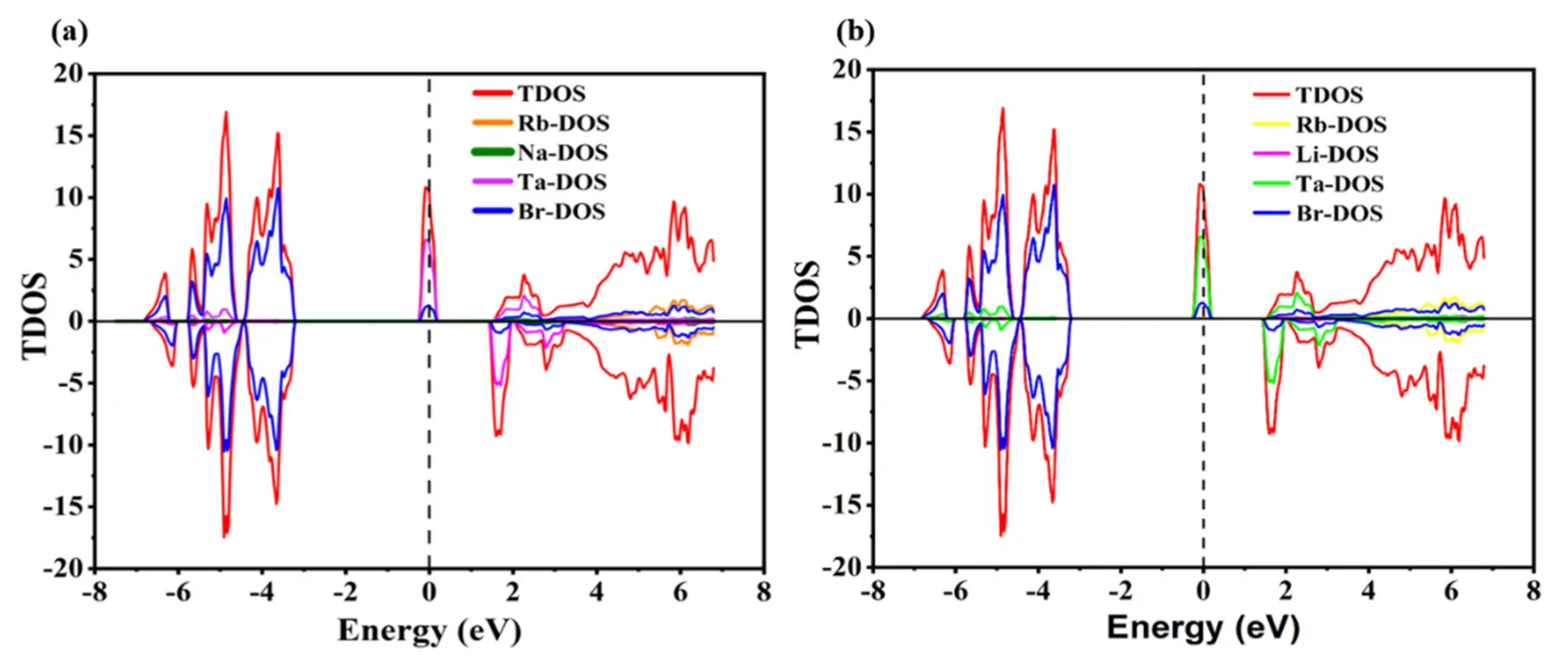
Spin Polarized TDOS of (**a**) Rb_2_NaTaBr_6_ and (**b**) Rb_2_LiTaBr_6_ calculated using GGA + U.

Electronic structure calculations were performed using GGA + U to treat on-site Coulomb interaction on the Ta-d orbitals. The spin-polarized band structures along the high-symmetry path W-L-Γ-X-W-K show multiple bands crossing the Fermi level (E_F_ = 0 eV) in both spin channels for both compounds, confirming intrinsic metallic character with zero band gap (Figure 3). This is corroborated by the total density of states (TDOS), which is finite at E_F_ for both compounds (10–11 states/eV/f.u.), consistent with metallic conduction. Partial density of states (PDOS) analysis in Figure 4 shows the Ta-d orbitals dominate the states near E_F_, serving as the primary conduction channel (Figure S1), while the valence band from −6 to −2 eV is dominated by Br-p states strongly hybridized with Ta-d, indicating pronounced Ta-Br covalency. Rb and Na/Li states contribute negligibly at E_F_, consistent with their ionic character. The PDOS of Rb_2_LiTaBr_6_ is broader near E_F_ than that of Rb_2_NaTaBr_6_, reflecting its wider metallic band (see below), i.e., more delocalized states. The Hubbard U correction sharpens the localization and energetic placement of the Ta-d states without opening a gap, confirming that the metallic character is strong to the U treatment (Section 2).

Both compounds are spin-polarized half-metallic ferromagnets. In the spin-up channel, a single band crosses E_F_ with a narrow dispersion of −0.35 to +0.10 eV in Rb_2_NaTaBr_6_ (0.45 eV) and −0.5 to +0.3 eV in Rb_2_LiTaBr_6_ (0.8 eV), confirming a genuine, narrow metallic channel in this spin direction for both compounds. In the spin-down channel, the lowest unoccupied band lies 0.85–0.9 eV above E_F_, so this channel is locally gapped in both compounds. This spin-up-metallic/spin-down-gapped combination is the electronic signature of half-metallic ferromagnetism and is what enforces the integer total magnetic moments obtained: 2.00089 μB (Rb_2_NaTaBr_6_) and 1.99995 μB (Rb_2_LiTaBr_6_) per formula unit (Table 2), each dominated by the Ta site (1.333 and 1.349 μB, respectively). The alkali metals (Rb, Na, Li) carry negligible moments, consistent with their non-magnetic ionic character; slight negative moments at the Li/Na and Br sites indicate weak antiparallel spin polarization from hybridization, and the interstitial region contributes the remainder of the total moment. Across the full investigated volume range, the ferromagnetic (FM) phase yields consistently lower total energy than the antiferromagnetic (AFM) counterpart for both materials, confirming ferromagnetism as the true ground-state ordering and excluding single-volume artifacts [30,31]. The FM-AFM energy stabilization is substantial in both cases, increasing from ΔE(AFM-FM) 0.081 Ry (1.10 eV) for Rb_2_LiTaBr_6_ to 0.116 Ry (1.58 eV) for Rb_2_NaTaBr_6_.

Inclusion of spin–orbit coupling (SOC) in the electronic-structure calculations for Rb_2_LiTaBr_6_ and Rb_2_NaTaBr_6_ reveals two clearly distinct regimes of relativistic response. The broad 5 eV separation between the Br-4p-dominated valence manifold (−3.1 to −3.3 eV) and the main Ta-5d conduction manifold (+1.7 to +2.1 eV) is essentially invariant under SOC, confirming that this large-scale electronic structure is governed by chemical bonding and crystal-field splitting rather than relativistic effects [32]. In sharp contrast, the narrow Ta-5d t_2_g band pinned at the Fermi level, weakly dispersive (0.3–0.4 eV) in the scalar-relativistic calculation, undergoes pronounced SOC-induced reshaping: it acquires strong k-dependent dispersion and oscillates across E_F_ along the high-symmetry path, directly reflecting the strong ξ L·S mixing of orbital and spin degrees of freedom for Ta (Z = 73). Despite this substantial modification of the near-Fermi-level states, SOC alone does not open a full electronic gap in either compound, and both systems retain a narrow-band metallic character. The total magnetic moment (spin + orbital) extracted from the SOC calculation is consistent with the 2 μB/f.u. obtained from the scalar-relativistic spin-polarized calculation, with the orbital contribution found to be small relative to the spin part.

**Figure 5:**
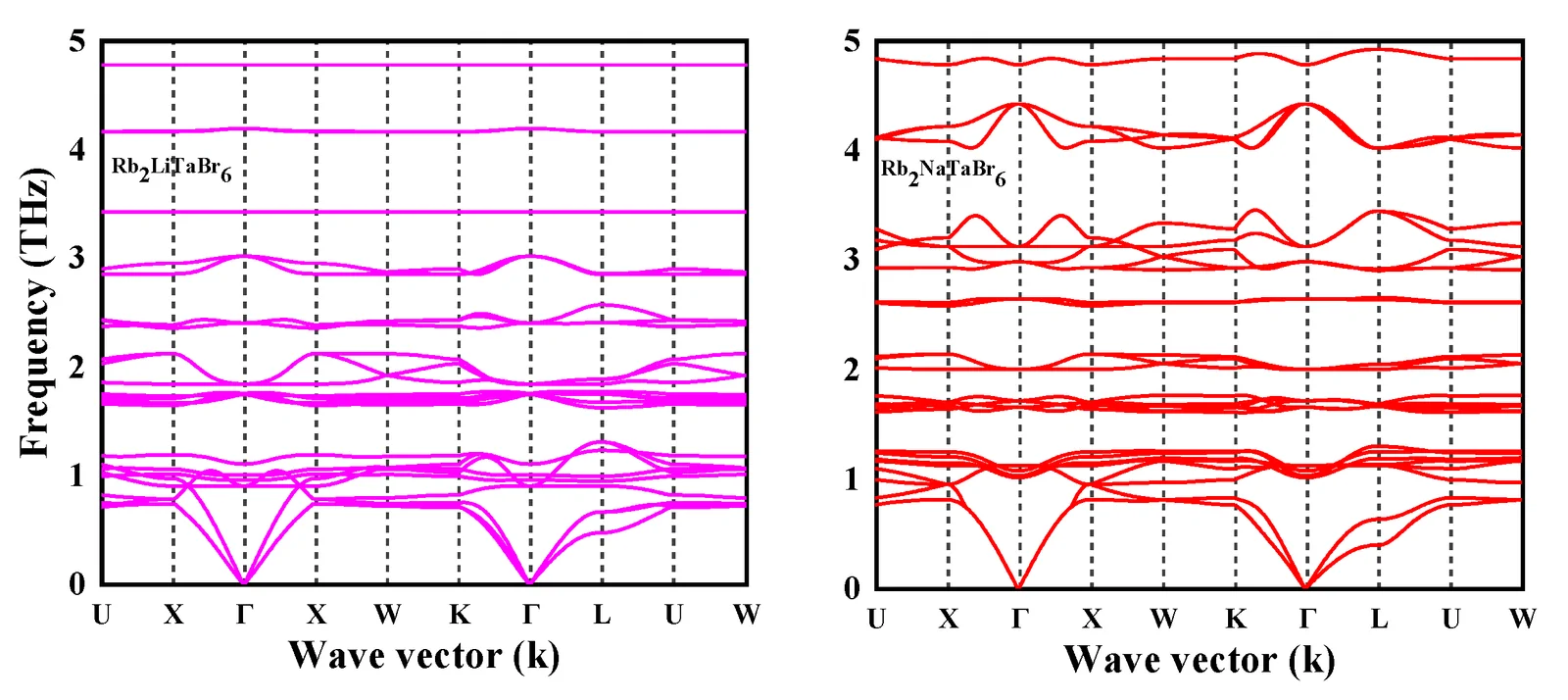
Phonon dispersion of Rb_2_LiTaBr_6_ and Rb_2_NaTaBr_6_.

The phonon dispersions (Figure 5) along the high-symmetry path U–X–Γ–X–W–K–Γ–L–U–W for both compounds, observing no imaginary frequencies across the Brillouin zone, which verifies dynamical stability of their cubic Fm-3m phases beyond mechanical stability from Born elastic criteria. Several flat optical phonon branches are identified, corresponding to localized rattling of alkali cations and weakly coupled TaBr_6_ octahedral vibrations, a characteristic feature of double-perovskite lattices.

## 4. Optical Properties

Rb_2_NaTaBr_6_ and Rb_2_LiTaBr_6_ exhibit strong interband absorption in the ultraviolet, originating from Br-p → Ta-d transitions. The calculated optical spectra include only interband contributions; the intraband Drude term was not computed, so the near-zero absorption below 3.5 eV reflects this omission rather than a semiconducting band gap. This omission is confined to the low-energy region: because the Drude and interband contributions to ε(ω) are additive and spectrally separable within the independent-particle (RPA) formalism implemented in WIEN2k OPTIC, it affects only the gap below the plasma energy ħωp and leaves the interband response above it unchanged. Given the intrinsic metallic ground state and this UV-centered interband response, these compounds are unsuitable for conventional visible-light photovoltaics but show potential for UV-focused optoelectronic applications such as photodetectors.

### 4.1. Dielectric Properties

**Figure 6:**
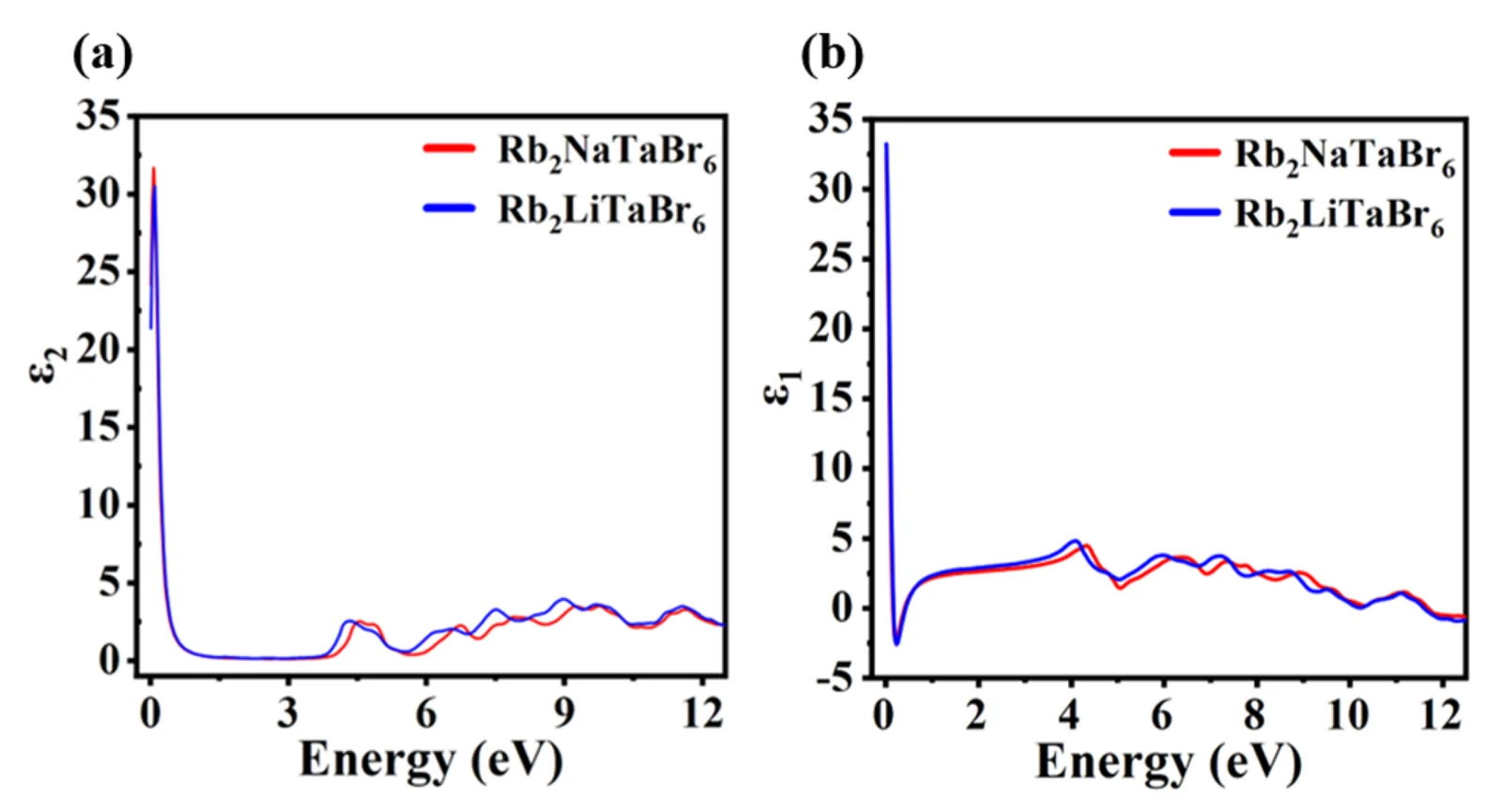
(**a**) Real part and (**b**) imaginary part of Rb_2_XTaBr_6_ (X = Na, Li).

The real (ε_1_) and imaginary (ε_2_) parts of the dielectric function for Rb_2_NaTaBr_6_ and Rb_2_LiTaBr_6_, shown in Figure 6a,b, characterize their optical polarization and absorption response. Both compounds show a very high static dielectric constant, ε_1_(0) = 33, indicating strong polarizability. ε_1_ then drops sharply, dipping negative between 0.3 and 0.8 eV (minimum = −2.5 to −3), rises through a broad hump (ε_1_ = 2–3) across 1–3 eV, peaks at ε_1_ = 4.5–5 near 4 eV—the onset of Br-p → Ta-d interband transitions—then oscillates downward through a weaker feature near 6–7 eV (ε_1_ = 3.5–4) before crossing negative again above 10 eV. ε_2_ shows a sharp low-energy peak (31–32 for both compounds) that collapses to near zero by 1 eV and stays flat through 3.5 eV, confirming weak absorption in this range rather than any semiconducting character. Absorption resumes above 4 eV with peaks near 4.3 eV (ε_2_ = 2.5–3), 7.5 eV (ε_2_ = 2–2.3), and most strongly near 9.3 eV (ε_2_ = 3.5–4), with Rb_2_LiTaBr_6_ running roughly 10–15% higher than Rb_2_NaTaBr_6_ across this 4–12 eV interband region.

### 4.2. Optical Conductivity

The optical conductivity of Rb_2_NaTaBr_6_ and Rb_2_LiTaBr_6_ is calculated up to 14 eV. The optical conductivity of both compounds is almost zero below about 4.0 eV, which reflects the absence of interband transitions in this range and does not establish semiconducting character because the intraband Drude term was omitted. At about 4.0–4.3 eV, a significant increase can be observed, which means that the interband electronic transitions take place. Above the energy of approximately 6.5 eV, there is strong optical response with several strong peaks seen in the 7.0–11.0 eV energy range, which is caused by increased interband transitions. Optical conductivity is highest in the 11.0–11.5 eV range with an intensity of Rb_2_LiTaBr_6_ being more intense than Rb_2_NaTaBr_6_, which has lower intensity. At higher energies, above 12 eV, the optical conductivity of both materials tends to decrease gradually. Altogether, the optical activity of both types of double perovskites is evident in the ultraviolet range, thus indicating their potential in the UV optoelectronics.

**Figure 7:**
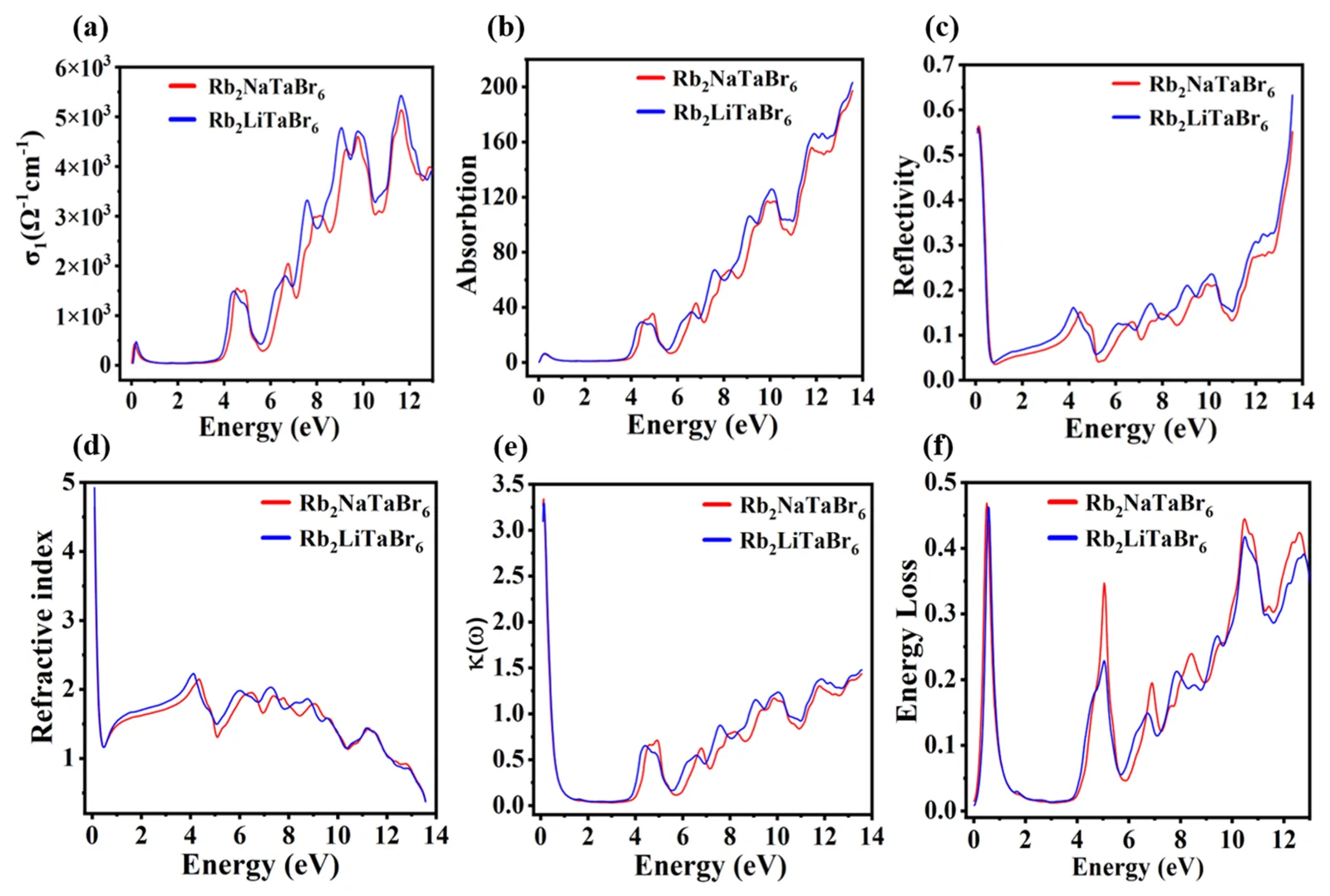
(**a**) Optical conductivity σ(ω), (**b**) absorption coefficient α(ω), (**c**) reflectivity R(ω), (**d**) refractive index n(ω), (**e**) extinction coefficient k(ω), and (**f**) electron energy-loss function L(ω) of Rb_2_XTaBr_6_ (X = Na, Li), as functions of photon energy (eV).

Figure 7 presents the optical response of Rb_2_NaTaBr_6_ and Rb_2_LiTaBr_6_ across 0–14 eV. The optical conductivity, σ_1_(ω) (Figure 7a), remains low below 3.5 eV, then rises through a series of interband peaks toward higher energy, with Rb_2_LiTaBr_6_ exceeding Rb_2_NaTaBr_6_ across the UV region. The absorption coefficient (Figure 7b) follows the same trend, negligible below 3.5 eV, consistent with the interband-only optical property established in Section 4, and rising steadily into the UV, confirming strong absorption in both compounds only once interband transitions become available. Reflectivity (Figure 7c) is high near zero energy, consistent with their metallic character, falls to a minimum once intraband screening is no longer probed at this energy scale, then rises again through the mid-energy range before increasing sharply at the highest energies as new interband channels open. The refractive index (Figure 7d) is nearly indistinguishable between the two compounds throughout the spectrum, indicating that Na/Li substitution has little effect on bulk polarizability even though it measurably changes the lattice constant and bonding strength. The extinction coefficient, κ(ω) (Figure 7e), tracks the absorption edge closely, with Rb_2_LiTaBr_6_ remaining consistently higher across the UV region. The energy-loss function (Figure 7f) shows the clearest departure from this pattern: Rb2NaTaBr6 exhibits a markedly stronger peak in the mid-energy range, before the two compounds converge again at higher energy.

## 5. Thermoelectric Properties

**Figure 8:**
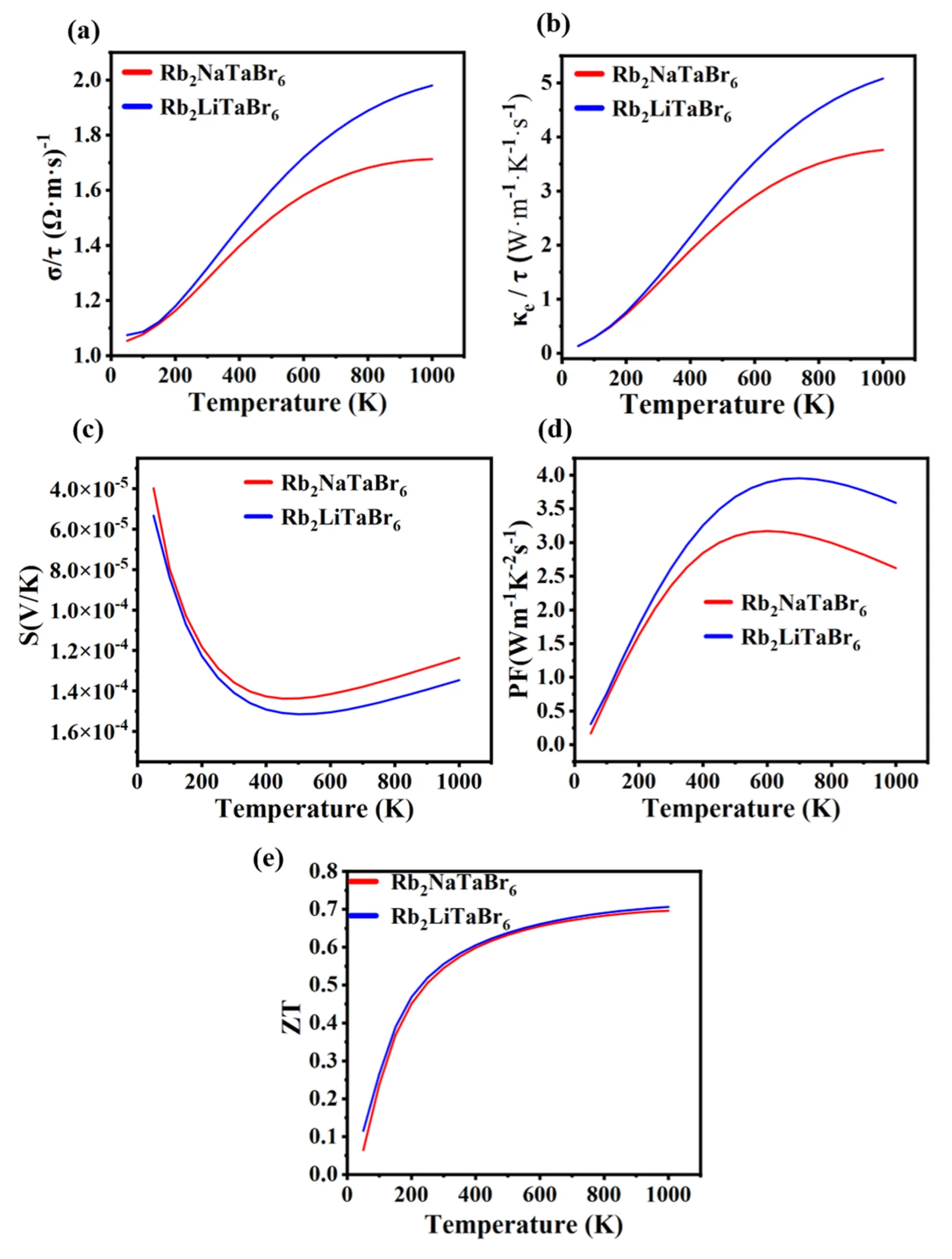
(**a**) Electrical conductivity, (**b**) thermal conductivity, (**c**) Seebeck coefficient, (**d**) power factor, and (**e**) figure of merit of Rb_2_XTaBr_6_ X = (Na, Li), double perovskites.

Rb_2_NaTaBr_6_ and Rb_2_LiTaBr_6_ were evaluated using temperature-dependent transport coefficients calculated within the constant relaxation-time approximation, as implemented in BoltzTraP. In this framework, BoltzTraP outputs the electrical conductivity and electronic thermal conductivity scaled by the relaxation time (σ/τ, in (Ω·m·s)^−1^, and κ_e_/τ, in W/(m·K·s)). The Seebeck coefficient S (V/K), by contrast, comes out independent of τ and needs no further assumption, getting an absolute electrical conductivity, and from it the power factor (PF = S^2^σ, in W/(m·K^2^)) and ZT plotted in Figure 8. We used τ = 1 × 10^−14^ s for Figure 8, the standard order-of-magnitude choice in halide-perovskite thermoelectric work when experimental scattering data are not available. With that value fixed, the calculated σ/τ, κ/τ, S, PF, and ZT point to metallic, electron-dominated transport under the conditions modeled here (Figure 8). Electrical and thermal conductivity rise with temperature in both compounds as carrier transport improves, and Rb_2_LiTaBr_6_ runs higher throughout thanks to better carrier mobility and more delocalized electronic states. The Seebeck coefficient stays negative across the whole temperature range, consistent with electron-like carriers dominating transport, and it falls as temperature rises, though Rb_2_NaTaBr_6_ holds a slight edge over Rb_2_LiTaBr_6_. Power factor peaks between 600 and 700 K, where conductivity and Seebeck coefficient strike their best balance, and Rb_2_LiTaBr_6_ again comes out ahead. ZT climbs steadily and reaches about 0.7 at 1000 K (Supplementary Materials), which counts as moderate thermoelectric performance rather than an exceptional one. Between the two, Rb_2_LiTaBr_6_ looks like the stronger candidate for high-temperature applications such as waste heat recovery and energy harvesting.

## 6. Conclusions

First-principles calculations were used to examine the structural, elastic, electronic, magnetic, optical, and thermoelectric behavior of Rb_2_NaTaBr_6_ and Rb_2_LiTaBr_6_. Structural relaxation produced local minima within the assumed cubic Fm-3m model, and the elastic constants satisfy the Born criteria, establishing mechanical stability under that symmetry. The analysis of the electronic structure demonstrates that it has a metallic nature with the Ta-d orbitals as the most significant at the Fermi level, which guarantees high electrical conductivity. The optical properties exhibit high dielectric response and strong absorption within the ultraviolet region, which makes them promising for ultraviolet optoelectronic components such as UV photodetectors. Nevertheless, their intrinsic metallic nature precludes their deployment in conventional visible-light photovoltaic solar cells that require a semiconducting band gap.

The BoltzTraP results indicate electron-dominated transport and show that Rb_2_LiTaBr_6_ generally has larger σ/τ and PF/τ trends than Rb_2_NaTaBr_6_. The electronic-only figure ZT_e_ = 0.7 at 1000 K, but this is an upper-bound indicator because lattice thermal conductivity was not included and should not be described as a realistic total ZT. The absolute conductivity, power factor, and ZT shown in Figure 7 rely on an assumed relaxation time (τ = 1 × 10^−14^ s), so these absolute values should be read as order-of-magnitude estimates rather than experimentally validated quantities. Moreover, SOC was omitted even though Ta-d states dominate near E_F_, so the quantitative thermoelectric trends require future GGA + U + SOC validation before strong performance claims can be made.

As a limitation, the intraband (Drude) contribution to the optical response was not computed in this work; based on the narrow spin-up band dispersion crossing E_F_ (−0.35 to +0.10 eV) and the TDOS(E_F_) values reported here (10–11 states/eV/f.u.), the associated plasma energy is expected to confine this correction to below 4 eV, leaving the UV interband application claims made in this work unaffected. A quantitative estimate of the plasma frequency and relaxation time, using the carrier-concentration and effective-mass data already generated in our BoltzTraP transport calculations, is identified as necessary future work to fully characterize the sub-gap optical response of these compounds.

## Figures and Tables

**Table 1:** Optimized structural parameters for Rb_2_NaTaBr_6_ and Rb_2_LiTaBr_6_ double-perovskite compounds.

Parameters	Rb_2_NaTaBr_6_	Rb_2_LiTaBr_6_
a_0_ (Å)	11.08	10.80
V_0_ (a.u^3^)	2275.5202	2101.1911
B (GPa)	27.6565	30.1903
B′	4.5470	4.7828

**Table 2:** Spin magnetic moment of mixed charge density in Rb_2_LiTaBr_6_ and Rb_2_NaTaBr_6_.

Compound Rb_2_LiTaBr_6_	Spin Magnetic Moments (µ_B_)	Compound Rb_2_NaTaBr_6_	Spin Magnetic Moments (µ_B_)
Interstitial	0.65238	Interstitial	0.66892
Rubidium	0.00176	Rubidium	0.00181
Lithium	−0.00182	Sodium	−0.00153
Tantalum	1.34904	Tantalum	1.33285
Bromine	−0.00053	Bromine	−0.00050
Total	1.99995	Total	2.00089

## Data Availability

The raw data supporting the conclusions of this article will be made available by the authors on request.

## Supplementary Materials

The following supporting information can be downloaded at: https://www.mdpi.com/article/10.3390/nano16181153/s1, Figure S1: Spin Polarized TDOS and PDOS of Rb_2_NaTaBr_6_ and Rb_2_LiTaBr_6_ calculated using GGA+U. Reference [33] is cited in the Supplementary Materials.
