# Supplementary material for: Multifunctionality in Lead-Free Rb2XTaBr6 (X = Na, Li) Double Perovskites: A First-Principles Study of Structural, Elastic, Optoelectronic, and Thermoelectric Properties
Source: Nanomaterials (Basel). 2026 Sep 14;16(18):1153. doi: 10.3390/nano16181153 (PMC13610120; doi:10.3390/nano16181153)
Supplement: Supplementary file 1 [file nanomaterials-16-01153-s001.zip › nanomaterials-4532586-supplementary.pdf]

1. Comparison of structure optimized parameters of Rb_2X_6Br (Na, Li) and K_2TlAsX_6 (Cl, Br)[1]

The optimized lattice constants of $Rb_2NaTaBr_6$ (11.08 Å) and $Rb_2LiTaBr_6$ (10.80 Å) are comparable to those of $K_2TlAsCl_6$ (11.00 Å) and $K_2TlAsBr_6$ (11.52 Å). The bulk moduli of $Rb_2NaTaBr_6$ (27.66 GPa) and $Rb_2LiTaBr_6$ (30.19 GPa) exceed those of $K_2TlAsCl_6$ (24.00 GPa) and $K_2TlAsBr_6$ (20.31 GPa), demonstrating enhanced resistance to volume compression. The pressure derivative of the bulk modulus B′ is similar for all four compounds, ranging from 4.547 to 4.865. Collectively, the Rb-based double perovskites, especially $Rb_2LiTaBr_6$, exhibit superior compressive stiffness relative to the K_2TlAsX_6 reference family.

2. Investigated elastic constant values for the Rb_2XTaBr_6 compounds with X = (Na, Li).[1]

The elastic constants of $Rb_2NaTaBr_6$ and $Rb_2LiTaBr_6$ are compared with reference double perovskites $K_2TlAsCl_6$ and $K_2TlAsBr_6$. The values of $\mathbf{C_{11}}$ for $Rb_2NaTaBr_6$ (61.83 GPa) and $Rb_2LiTaBr_6$ (59.04 GPa) exceed those of $K_2TlAsCl_6$ (55.43 GPa) and $K_2TlAsBr_6$ (43.47 GPa), indicating stronger resistance to longitudinal deformation. Similarly, both Rb-based compounds show larger C_{44} values (10.19 and 11.08 GPa) than the reference compounds (5.05 and 6.19 GPa), suggesting enhanced resistance to shear deformation. Their Young's moduli are also higher or comparable, while the B/G ratios of 3.08 and 3.68 exceed those of $K_2TlAsCl_6$ (3.04) and $K_2TlAsBr_6$ (2.14), indicating a more pronounced ductile nature. Collectively, $Rb_2NaTaBr_6$ and $Rb_2LiTaBr_6$ exhibit superior elastic stiffness and enhanced ductility compared with the K_2TlAsX_6 reference family.

3. Comparative Thermoelectric Properties of Rb_2XTaBr_6 (X= Na, Li)[1]

The thermoelectric properties of $Rb_2NaTaBr_6$ and $Rb_2LiTaBr_6$ were compared with those of $K_2TlAsCl_6$ and $K_2TlAsBr_6$. Both Rb-based compounds exhibit lower electrical conductivity but favorable thermoelectric performance at elevated temperature. At 1000 K, $Rb_2NaTaBr_6$ and $Rb_2LiTaBr_6$ show ZT values of 0.71 and 0.69, respectively, which are comparable to those of $K_2TlAsCl_6$ (0.75) and $K_2TlAsBr_6$ (0.75). Among the studied Rb-based compounds, $Rb_2NaTaBr_6$ exhibits slightly superior performance, with higher Seebeck coefficient, power factor, and ZT, indicating its promising potential for high-temperature thermoelectric applications.

Spin-polarized partial density of states (PDOS) calculations, performed within the GGA+U framework, are shown for $Rb_2NaTaBr_6$ and $Rb_2LiTaBr_6$ in Figure. S1a, b, respectively. In both compounds, the valence band is dominated by Br *p* orbitals and the conduction band minimum by Ta *d* states. Rb contributes negligibly across the entire energy range, consistent with its role as an electronically inert A-site cation. In $Rb_2NaTaBr_6$, Na-derived states are essentially absent near the band edges. By contrast, in $Rb_2LiTaBr_6$, distinct Li *s* and *p* states appear within the valence band, indicating that the smaller Li^+ cation participates in valence-band orbital mixing, a feature absent in the Na analogue. Br *s* states lie deep in the valence band, well below the Fermi level, whereas Br *d* and *f* states carry negligible spectral weight in the plotted range. The Ta *d* character of the conduction band edge is preserved in both compounds, indicating that the octahedral Ta framework governs the low-energy conduction states irrespective of whether Na or Li occupies the A site. Thus, substitution of Na^+ by Li^+ alters the valence-band orbital composition through the emergence of Li *s/p* states, while the fundamental band-edge character, Br *p* at the valence-band maximum and Ta *d* at the conduction-band minimum, remains unchanged.

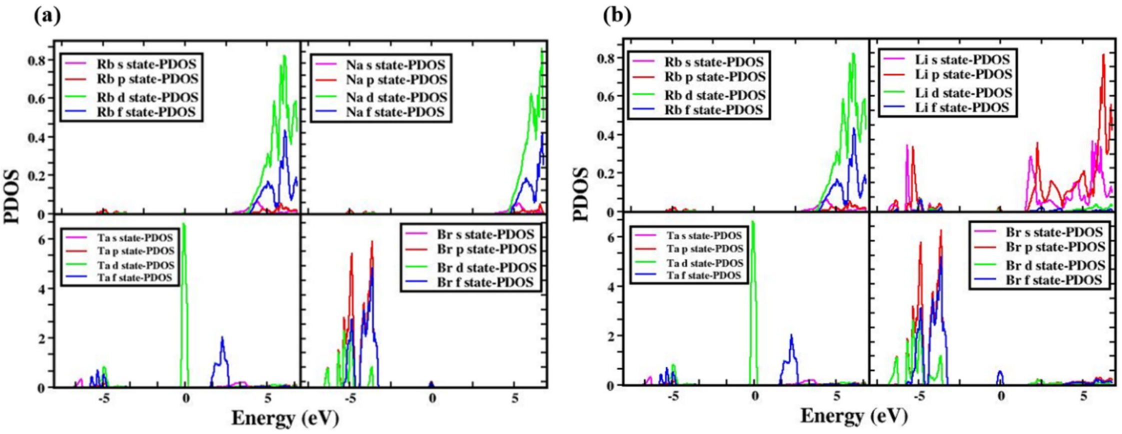

Figure. S1. Spin Polarized TDOS and PDOS of $Rb_2NaTaBr_6$ and $Rb_2LiTaBr_6$ calculated using GGA+U.

Reference

[1]. Munir, J., et al., First-principles investigations of the mechanically and thermodynamically stable potassium-based double perovskites K_2TlAsX_6 (X= Cl, Br) for optoelectronic and renewable applications, Materials Science and Engineering: B, 2023, 298 116830
